# Two-dimensional BiTeI as a novel perovskite additive for printable perovskite solar cells†

**DOI:** 10.1039/d2se01109c

**Published:** 2022-10-24

**Authors:** Dimitris Tsikritzis, Konstantinos Chatzimanolis, Nikolaos Tzoganakis, Sebastiano Bellani, Marilena Isabella Zappia, Gabriele Bianca, Nicola Curreli, Joka Buha, Ilka Kriegel, Nikolas Antonatos, Zdeněk Sofer, Miron Krassas, Konstantinos Rogdakis, Francesco Bonaccorso, Emmanuel Kymakis

**Affiliations:** a Department of Electrical & Computer Engineering, Hellenic Mediterranean University (HMU) Heraklion 71410 Crete Greece kymakis@hmu.gr; b Institute of Emerging Technologies (i-EMERGE) of HMU Research Center Heraklion 71410 Crete Greece; c BeDimensional S.p.A. Via Lungotorrente Secca 30R 16163 Genova Italy; d Graphene Labs, Istituto Italiano di Tecnologia via Morego, 30 16163 Genova Italy; e Functional Nanosystems, Istituto Italiano di Tecnologia via Morego, 30 16163 Genova Italy; f Department of Nanochemistry, Istituto Italiano di Tecnologia via Morego, 30 16163 Genova Italy; g Department of Inorganic Chemistry, University of Chemistry and Technology Prague Technická 5 Prague 6 16628 Czech Republic

## Abstract

Hybrid organic–inorganic perovskite solar cells (PSCs) are attractive printable, flexible, and cost-effective optoelectronic devices constituting an alternative technology to conventional Si-based ones. The incorporation of low-dimensional materials, such as two-dimensional (2D) materials, into the PSC structure is a promising route for interfacial and bulk perovskite engineering, paving the way for improved power conversion efficiency (PCE) and long-term stability. In this work, we investigate the incorporation of 2D bismuth telluride iodide (BiTeI) flakes as additives in the perovskite active layer, demonstrating their role in tuning the interfacial energy-level alignment for optimum device performance. By varying the concentration of BiTeI flakes in the perovskite precursor solution between 0.008 mg mL^−1^ and 0.1 mg mL^−1^, a downward shift in the energy levels of the perovskite results in an optimal alignment of the energy levels of the materials across the cell structure, as supported by device simulations. Thus, the cell fill factor (FF) increases with additive concentration, reaching values greater than 82%, although the suppression of open circuit voltage (*V*_oc_) is reported beyond an additive concentration threshold of 0.03 mg mL^−1^. The most performant devices delivered a PCE of 18.3%, with an average PCE showing a +8% increase compared to the reference devices. This work demonstrates the potential of 2D-material-based additives for the engineering of PSCs *via* energy level optimization at perovskite/charge transporting layer interfaces.

## Abstract 2

2D BiTeI flakes were exfoliated and incorporated in PSCs as an additive in the perovskite layer. The energy level alignment changes from “cliff” to “spike” upon BiTeI incorporation. The FF increases to 82% and the PCE to 18.3%.

## Graphical abstract

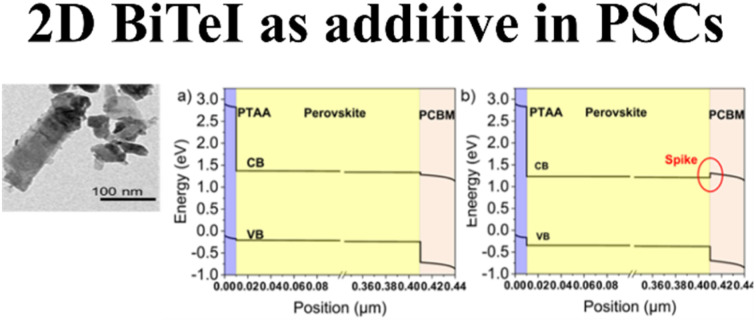

## Introduction

The power conversion efficiency (PCE) of perovskite solar cells (PSCs) has been continuously increasing for the last 10 years to more than 25%,^1,2^ approaching the record-high values of Si-based solar cells.^3^ Despite the promise of PSCs, it is still crucial to solve the remaining challenges related to stability,^4,5^ performance reproducibility, and reliability,^6,7^ which call for the urgent exploration of novel non-complex and cost-effective manufacturing procedures.^8–10^ The optimization of the charge transporting layers at the interfaces with the perovskite is a prototypical route to tune the material energy alignment across the cell structure so that favorable material energy offsets can lead to PCE maximization.^11–13^ Recently, a major breakthrough in PSC technology was achieved by the demonstration of the world's first solar farm enabled by perovskite solar panels incorporating two-dimensional (2D) materials, paving the way for PSC commercialization.^14^ In inverted PSCs, the perovskite shows a p-type character,^15^ while its conduction (CB) and valence band (VB) energy levels offer suitable energy alignment at NiO/perovskite and perovskite/C_60_ interfaces, respectively.^16^ However, inadequate energy level offsets at the perovskite interface with 2,2′,7,7′-tetrakis[*N*,*N*-di(4-methoxyphenyl)amino]-9,9′-spirobifluorene (spiro-MeOTAD)^17,18^ and poly(triaryl amine) (PTAA)^19^ result in unoptimized device performance when these efficient organic hole transport layers are used. The doping/modification of the perovskite active layer with molecules or other types of additives is a valuable engineering approach for preparing high-PCE and stable PSCs.^20–22^ Specifically, recent studies have demonstrated the beneficial role of incorporating various additives into the perovskite, showing improvements in terms of crystallization of the perovskite layer,^20,21^ increase of the grain size,^23,24^ and suppression of the trap density.^25,26^ Such effects enhanced the environmental stability of PSCs,^27,28^ while introducing complementary smart functionalities (*e.g.*, self-healing properties) of the materials.^29,30^ Perovskite doping with metal cations has been reported to result in n- or p-type doping that could promote energy level alignment, leading to interesting device performances.^31^ Notably, the incorporation of Sr, Cu, Sb, Bi, Mg, and Ce into the perovskite resulted in n-doping,^22,32–36^ while p-type doping was achieved using Ag^37,38^ and Mo-based oxidants.^39^ Recently, perovskite has also been modified by the incorporation of 2D layered materials, including graphene^40^ and MXenes,^41–43^ both of which exhibit metallic behavior. This novel class of additives enabled fine-tuning of the work function (*W*_F_) of the perovskite absorber and suppression of ion migration effects, leading to substantial performance improvements in 2D material-engineered PSCs. The exploration of other types of 2D materials as perovskite additives with different optoelectronic properties compared to graphene and MXenes represents a potential approach to allow further optimization of PSCs by achieving optimal energy level alignment at perovskite/charge transporting layer interfaces.

In this context, bismuth telluride iodide (BiTeI) is a layered polar semiconductor that is formed by loosely coupled atomic layers of Bi sandwiched between Te and I, while the tri-layer blocks are stabilized by van der Waals bonds. The mixed covalent-ionic characters of BiTeI, modeled as ionically bound (BiTe)^+^ and I^−^ layers,^44,45^ together with the presence of heavy Bi atoms, result in a non-centrosymmetric structure with sizeable spin–orbit interaction (SOI) effects,^46^ namely giant Rashba effect.^47,48^ BiTeI has a narrow bandgap of 0.3–0.7 eV (ref. 49 and 50), while its dielectric constant was measured in the range of 10–12 (ref. 51) and the refractive index is between 4 and 2.5 in the visible region.^52^ Theoretical calculations have shown that a 0.289 eV Å^−1^ net electric field points from the (BiTe)^+^ to the I^−^ layers, as a result of a partial charge transfer of 0.380 and 0.390 e^−^ from Bi to Te and Bi to I, respectively.^53^ The carrier concentration of BiTeI was estimated to be 0.2–7 × 10^19^ cm^−3^, while the calculated electron effective mass is ∼0.2 m_e_ and 1 m_e_ for the *ab* plane and *c* axis, respectively.^54^ In ref. 53, the calculated effective mass of the carriers was 0.176 m_e_ for electrons and 0.537 for holes, while the electron and hole mobilities (*μ*_e_ and *μ*_h_) were 392.34 cm^2^ V^−1^ s^−1^ and 46.55 cm^2^ V^−1^ s^−1^, respectively. The conductivity of BiTeI mainly originates from free electrons, and the carrier concentration strongly depends on the crystal growth conditions.^55^ Nevertheless, the existence of n- and p-carriers at the 2D plane of the BiTeI surfaces (Te-rich and I-rich domains, respectively) allows ambipolar conduction,^56^ which may be desirable in photovoltaic devices. The charged surfaces of BiTeI induce a surface band bending in the near-surface layers,^57^ which can lead to interfacial effects related to charge extraction. For surface and subsurface layers, defects can significantly modify the electronic states in the I-terminated region (by more than 100 meV energy), while the states in the Te-terminated surface areas remained unmodified.^58^ Beyond creating finite out-of-plane dipole moments and strong SOI,^59,60^ the peculiar structures of BiTeI also lead to the emergence of piezoelectricity properties for mechanical-to-electrical energy conversion devices.^61^ Other examples of applications of BiTeI are thermoelectric devices,^48^ spintronics,^45^ catalysis (*e.g.*, nitrogen reduction reaction^62^) and non-linear optics.^63^ By taking advantage of the natural cleavage planes offered at the interface between Te and I, the exfoliation of BiTeI in 2D forms is a turning point to fully exploit its surface properties,^60,63^ as well as its integration as an additive in thin films of functional materials, including metal halide perovskites of PSCs. The predicted cleavage energy of BiTeI to obtain its monolayer is as low as ∼90 meV per atom,^63^ which is of the same order as those reported for other layered materials, for example, metal chalcogenides^64–66^ and graphite.^67^ Single-/few-layer BiTeI flakes have been recently obtained through both mechanical^68^ and liquid-phase exfoliation (LPE) methods.^62,63^ For the former, stripped gold exfoliation was used to obtain for the first time BiTeI monolayers.^68^ However, such a methodology causes a strong hybridization of the BiTeI monolayer with the Au substrate, causing substantial modifications of the surface charge distribution on the BiTeI surface.^68^ Multi-layer BiTeI flakes with thicknesses up to 10 nm have recently been produced by electrochemical exfoliation using *N*,*N*-dimethylformamide solvent and tetrabutylammonium or lithium cations.^62^ However, these methods altered the structural properties of the flakes by inducing topological etching of the iodine atoms. The limitations of the previous exfoliation techniques have been recently solved by prototypical ultrasonication-assisted LPE, which preserved the structural characteristics of bulk BiTeI crystals (except for the layer number reduction).^63^ In particular, alcoholic solvents can minimize the Gibbs free energy of the solvent/BiTeI mixture, the primary condition to attain an efficient LPE of layered materials,^69,70^ leading to scalable production of BiTeI inks for large-scale, high-throughput manufacturing of devices, including solar cells.^71–73^

Owing to the metallic character of BiTeI, and by rationalizing the main characteristics of 2D materials previously used as additives for perovskites, we first evaluated the impact of LPE-exfoliated BiTeI flakes as additives for perovskite absorbers in inverted PSCs. Indeed, we show that 2D BiTeI additives in the active layer can regulate the material energy-level alignment. By varying the concentration of BiTeI flakes in the perovskite precursor solution between 0.008 mg mL^−1^ and 0.1 mg mL^−1^, combined experimental measurements and simulations demonstrated a downshift of the perovskite energy levels, adjusting the energy offsets at perovskite/charge transporting layer interfaces. Consequently, our 2D BiTeI-based engineering approach improves the performance of reference cells, achieving fill factor (FF) values above 82%. The optimum device configuration exhibited a PCE of 18.3%, with an average PCE showing a +8% increase compared to the reference devices. Our results confirm that metallic 2D materials can be effective additives for perovskite absorbers, encouraging further research on their use in photovoltaic devices.

## Experimental

The solvents were provided by Sigma Aldrich, PTAA by Solaris (*M*_w_ = 20–70 kDa), PbI_2_ and PbBr_2_ by TCI America, FAI (FA = formamidinium) and MABr (MA = methylammonium) by GreatCell Solar, CsI and RbI by Sigma Aldrich, PC_70_BM (6,6-phenyl-C71-butyric acid methyl ester, hereafter simply referred to as PCBM) by Solenne, and bathocuproine (BCP) by Sigma Aldrich. All solvents and commercial reagents were used as received unless otherwise stated.

## Crystal growth and exfoliation

The BiTeI crystals were grown by direct reaction of atomic elements (Bridgman method), following protocols previously reported in the literature.^62^ Briefly, stoichiometric amounts of Bi, Te, and I were placed in a quartz glass ampule and sealed under a high vacuum with an oxygen-hydrogen torch. To impede iodine loss, liquid N_2_ cooling was used during ampoule sealing. The ampule was then heated at 650 °C, using a 1 °C min^−1^ heating rate. After 6 h, it was cooled down to 400 °C with a cooling rate of 0.2 °C min^−1^. Finally, the so-obtained material was treated for 7 days at 400 °C and cooled down to room temperature. The as-growth BiTeI crystals were exfoliated through ultrasonication-assisted LPE.^74^ The unexfoliated material was discarded by means of sedimentation-based separation (SBS).^75^ Experimentally, 50 mg of powdered bulk crystals were mixed with 50 mL of anhydrous isopropanol (IPA) and ultrasonicated in a bath sonicator (Branson® 5800 cleaner, Branson Ultrasonics) for 15 h. The as-produced dispersions are ultracentrifuged at 700 g (Optima™ XE 90 with a SW32Ti rotor, Beckman Coulter) for 20 min at 15 °C. Then, 80% of the supernatant was collected by pipetting, obtaining the dispersions of the exfoliated materials. A solvent exchange process was then carried out to redisperse the BiTeI flakes in 4 : 1 vol./vol. anhydrous *N*,*N*-dimethylformamide (DMF):dimethyl sulfoxide (DMSO). Specifically, the dispersion of BiTeI flakes in IPA was ultracentrifuged at 5000*g* and the precipitate was redispersed in the DMF : DMSO mixture. The procedure was repeated three times, until obtaining a BiTeI flake dispersion in DMF:DMSO with a concentration of 0.2 mg mL^−1^.

## Materials characterization

Bright-field transmission electron microscopy (BFTEM) images of BiTeI flakes were acquired using a JEM 1400Plus (JEOL) TEM (thermionic source LaB6 crystal), operating at 120 kV. High-resolution TEM (HRTEM) measurements were carried out using a JEOL FSJEM2200 image-corrected microscope operated at 200 kV and equipped with an in-column Omega energy filter and Bruker Quantax 400 EDX system with a 60 mm^2^ XFlash detector. The samples for these observations are prepared by drop-casting the dispersion of the BiTeI flakes in DMF:DMSO onto ultrathin C-on-holey C-coated Cu grids or ultrathin C-coated Cu grids.

Atomic force microscopy (AFM) images were acquired by means of a NX10 AFM (Park System, Korea) by means of a Non-Contact Cantilever PPP-NCHR 10 M (Nanosensors, Switzerland) having a tip diameter smaller than 10 nm, a resonance frequency of ∼330 kHz, and a force constant of 42 N m^−1^. The images were collected in noncontact mode on an area of 5 × 5 μm^2^ (1024 × 1024 data points) keeping the working set point above 70% of the free oscillation amplitude. The scan rate for the acquisition of the images is 0.2 Hz. The samples are prepared by drop-casting a 1 : 10 diluted dispersion of BiTeI flakes in DMF:DMSO onto mica sheets (G250-1, Agar Scientific Ltd.) and heating to 100 °C for 1 h under vacuum to dry the sample and remove adsorbates.

XRD measurements were performed using a PANalytical Empyrean using Cu Kα radiation. Samples were prepared by depositing powder of BiTeI bulk crystals or BiTeI flakes (from a DMF:DMSO dispersion) on Si/SiO_2_ substrates.

Raman spectroscopy measurements were acquired using a Renishaw microRaman inVia 1000 mounting an objective with a 0.9 numerical aperture (NA), using an excitation wavelength of 514 nm and an incident power of 1 mW. For each sample, 50 spectra were collected for statistical purposes and to verify the reproducibility of the data. The samples were prepared by drop-casting the as-prepared BiTeI flake dispersion onto Si/SiO_2_ substrates and then dried under vacuum.

Gwyddion 2.60 software was used to process the height profiles of the flakes imaged by AFM, while ImageJ software (NIH) was used to analyze the lateral size of the flakes imaged by BFTEM. The lateral size of a flake was estimated as the mean of maximum and minimum lateral sizes of the flakes. OriginPro 9.1 software was also used to perform the statistical analysis of the thickness and lateral size data. 300 BiTeI flakes on multiple AFM and TEM images were considered for the statistical analysis of the thickness and lateral size, respectively, of the BiTeI flakes.

## Device fabrication

The complete fabrication process of PSCs has been described in detail in authors' previous work.^76^ Briefly, a thin film of PTAA was deposited from a 2 mg mL^−1^ solution on ITO substrates treated with UV-Ozone for 20 min and then annealed at 110 °C for 10 min. The quadruple cation perovskite precursor solution was prepared by dissolving 45.35 mg MABr, 394.7 mg FAI, 66.0 mg PbBr_2_, and 1266.4 mg PbI_2_ in 1.8 mL 4 : 1 vol./vol. anhydrous DMF : DMSO. Next, into the solution 114.2 μL and 95.2 μL from 1.5 M CsI and 1.5 M RbI stock solutions were added, respectively. The nominal composition of the quadruple perovskite was Cs_0.05_Rb_0.04_(FA_0.85_MA_0.15_)_0.91_Pb(I_0.91_Br_0.09_)_3_. To engineer the perovskite layer, BiTeI flakes were mixed in the perovskite precursor solution, varying their concentration between 0.008 mg mL^−1^ and 0.1 mg mL^−1^ through the addition of aliquots of the BiTei flake dispersion in 4 : 1 vol./vol. anhydrous DMF : DMSO. The perovskite layer was dynamically spin-coated onto ITO/PTAA substrates at 6000 rpm for 45 s. At 20 s before the end of the spinning process, 200 mL of anhydrous chlorobenzene (CB) was added dropwise onto the spinning perovskite film. Subsequently, the samples were immediately annealed for 45 min on a pre-heated hotplate at 100 °C. Next, an approximately 30 nm-thick layer of PCBM was spin-coated from a 20 mg mL^−1^ solution in anhydrous CB at 2000 rpm for 60 s. Subsequently, BCP was spin-coated from a 0.5 mg mL^−1^ solution prepared in extra dry IPA at 4000 rpm for 45 s. Finally, a 100 nm thick Ag top electrode was deposited by thermal evaporation under a high vacuum of 10^−6^ mbar.

## Device characterization and simulations

Steady-state and time-resolved photoluminescence measurements (PL, Tr-PL) were conducted using an FS5 spectrofluorometer (Edinburgh Instruments). A pulsed laser diode (*λ* = 478.4 nm, pulse full-width at half maximum 70 ps, and repetition rate 200 kHz to 40 MHz) was used to excite the samples. The VB energy position and the *W*_F_ of the perovskite films were estimated by ambient photoemission spectroscopy (APS) using an APS04 N2-RH system (KP Technology). The contact potential difference (CPD) was measured utilizing a gold alloy vibrating probe (2 mm diameter). The absolute *W*_F_ of the tip was estimated to be around 4.55 eV, which was calibrated by measuring an Ag reference and calculating its absolute *W*_F_ by APS. The material VB was determined using a UV light excitation source (D2) and by extrapolating to zero the cube root of the photoemission signal. The surface morphology and crystal structure of the perovskite films were investigated by field emission scanning electron microscopy (SEM) (JEOL 7000F) and the X-ray diffraction (XRD) patterns were collected using a Panalytical X’Pert PRO MPD system with CuKα radiation operated at 45 kV and 40 mA. A typical scan rate was 1 s per step with a step size of 0.02°.

The PSCs were evaluated under an inert atmosphere using an ABB solar simulator (Sol1A, Oriel), equipped with a 450 W Xe lamp and an AM1.5G filter. The intensity was calibrated at 100 mW cm^−2^ using a KG5 windowed Si reference cell. The *J*–*V* curves were recorded at a constant scan rate of 20 mV s^−1^ using a multiplexor test board system (Ossila), and no device preconditioning was applied prior to measurements. During each measurement, a black metallic aperture mask was used to set the active area of the manufactured devices at 0.04 cm^2^ and to reduce the influence of scattered light.

Device simulations were conducted with SCAPS-1D software.^77^ The input parameters used in the simulations were extracted by experimental routes and from the literature and are tabulated in Tables S1–S3.†

## Results

**Fig. 1:**
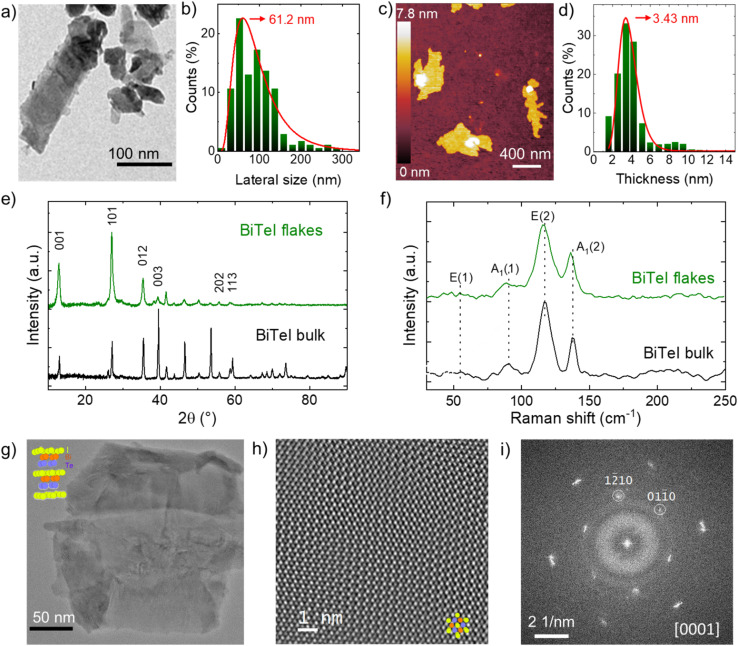
(a) BFTEM and (b) statistical analysis of the lateral size of the BiTeI flakes (300 flakes). (c) AFM images of and (d) statistical analysis of the thickness of the BiTeI flakes (300 flakes). (e) XRD patterns and (f) Raman spectra (excitation wavelength 514 nm) of the BiTeI bulk crystals and flakes. The panels also report the name of diffraction peaks and the Raman modes attributed to the hexagonal *P*3*m*1 structure of the BiTeI crystals. (g) HRTEM image of a representative BiTeI flake and (h) a close-up view showing the atomic arrangement as viewed from the [0001] direction. The atomic arrangement matches the expected atomic arrangement of BiTeI in the same orientation (a model is overlaid on top). (i) FFT of the HRTEM image confirming the single-crystalline nature of the flake and a [0001] orientation.

The BiTeI flakes were produced through ultrasonication-assisted LPE in IPA of hexagonal (space group *P*3*m*1, no. 156) BiTeI crystals, which were grown *via* direct synthesis of their elements.^62^ Importantly, IPA has a surface tension close to 28 mN m^−2^ (surface energy of 60 mJ m^−2^) and Hildebrand parameter between 19 and 25 MPa^0.5^. Such parameters' value/range minimizes the Gibbs free energy of the mixture solvent/BiTeI crystals, maximizing the exfoliation yield and stabilizing the exfoliated material dispersion.^63,78,79^ A solvent exchange treatment of the as-produced BiTeI dispersion was then carried out to redisperse the exfoliated materials in an anhydrous DMF : DMSO (4 : 1 vol./vol.) mixture, which was used to dissolve the quadrupole cation perovskite precursors.^80^Fig. 1a shows a BFTEM image of the BiTeI flakes, which have shapes with irregular jagged geometries. The statistical analysis of the lateral size of the BiTeI flakes (Fig. 1b) indicates that the data can be fitted with a log-normal distribution peaked at 61.2 nm and a maximum value approaching 300 nm. Fig. 1c shows an AFM image of representative BiTeI flakes. The statistical analysis of the thickness of the BiTeI flakes (Fig. 1d) reveals that the data follow a log-normal distribution peaked at ∼3.43 nm, which means that flakes are mainly single-/few-layer ones (experimental AFM thickness of the BiTeI monolayer is 8.5 ± 1.2 Å on an Au substrate^68^ and ∼1 nm on a mica substrate,^63^ while the bulk lattice parameters of BiTeI in the out-of-plane direction are reported to be in the 6.5–6.9 Å range).^44^Fig. 1e reports the XRD patterns of the BiTeI flakes and native bulk crystals, showing a matching with the hexagonal *P*3*m*1 structure (ICSD card 79364).^44,81^ Thus, the exfoliation process preserves the in-plane crystallinity of the sample, without leading to any additional phase (*e.g.*, oxides). The Raman spectra of both the BiTeI flakes and the bulk crystals (Fig. 1f) show the main modes predicted for the BiTeI crystals by group theory (four active Raman modes, with the irreducible vibrational representation *Γ* = 2*A*_1_ + 2*E*., *i.e.*, two *E* modes and two *A*_1_ modes).^82,83^ In particular, the Raman peaks at ∼90 and ∼138 cm^−1^ refer to *A*_1_(1) and *A*_1_(2) modes, respectively, and those at 58 and 117 cm^−1^ are ascribed to *E*(1) and *E*(2). The structural properties of the BiTeI flakes were further evaluated through HRTEM measurements. Fig. 1g reports a HRTEM image of a representative BiTeI flake. Fig. 1h shows a close-up view of the flake, revealing an atomic arrangement as viewed from the [0001] direction. The corresponding fast Fourier transform (FFT) of the HRTEM image (Fig. 1i) confirms the single-crystalline nature of the flake and an exact orientation [0001], indicating that the bulk BiTeI is exfoliated perpendicular to the *c* axis of its hexagonal crystal structure, as expected by its layered structure.

Upon verifying their successful exfoliation in 2D forms, the BiTeI flakes were then incorporated as functional additives into the perovskite layer, aiming at tuning the overall carrier concentration and charge transporting properties at the interfaces of perovskite with the charge transporting layers. It is noteworthy that the I-terminated surfaces of the BiTeI flakes could promote the interaction with the undercoordinated Pb atoms of the perovskite,^84^ reducing the trap density of the absorber layer. Thus, the BiTeI flakes were mixed in the perovskite precursor solution, varying their concentration between 0.008 mg mL^−1^ and 0.1 mg mL^−1^. Subsequently, six solutions were prepared and used for the fabrication of inverted PSCs (p-i-n) based on the ITO/PTAA/perovskite/PCBM/BCP/Ag configuration.

**Fig. 2:**
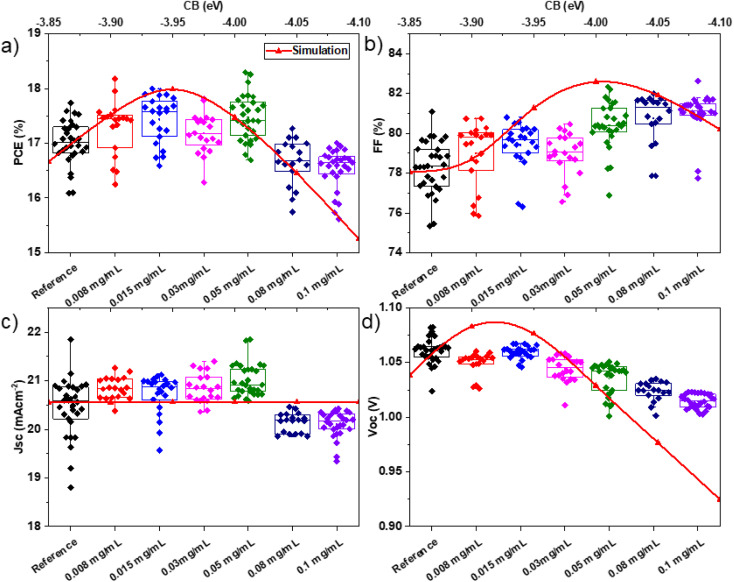
Photovoltaic characteristics of the PSCs produced using pristine perovskite (reference) and BiTeI incorporating perovskite, varying the BiTeI concentration in the perovskite precursor solution from 0.008 mg mL^−1^ up to 0.1 mg mL^−1^: (a) PCE, (b) FF, (c) *J*_sc_, and (d) *V*_oc_. The red line shows the simulation results acquired by varying the CB minimum energy level of perovskite from −3.85 to −4.1 eV with a step of 0.05 eV.

Fig. 2 shows the performance of the resulting devices, plotting the main photovoltaic metrics, *i.e.*, PCE, FF, short circuit current density (*J*_sc_), and open circuit voltage (*V*_oc_) (panels a–d, respectively). As shown in Fig. 2a, the addition of the BiTeI flakes with a concentration up to 0.05 mg mL^−1^ in the perovskite precursor solution increases the device PCE, reaching a maximum value of 18.3%. For these devices, the average PCE was 17.46%, which represents an increase of about +8% compared to the mean values of the reference devices. For a BiTeI flake concentration of 0.008 mg mL^−1^, the PSCs reached an average PCE of 17.45%, with the champion device reporting a PCE of ∼18.2%. By further increasing the number of the BiTeI flakes above a concentration of 0.08 mg mL^−1^, the device PCE started to deteriorate. Fig. 2b–d reveal the impact of the number of BiTeI flakes on the other device parameters (beyond the PCE). The FF and *V*_oc_ are the parameters that are most affected by the BiTeI concentration. As shown in Fig. 2b, FF increases monotonically with the concentration of BiTeI flakes, while *V*_oc_ shows the opposite trend. In particular, the mean *V*_oc_ decreased significantly at a BiTeI concentration higher than 0.03 mg mL^−1^, while the average FF increased from 78.3 in the reference device to 81.2% for the highest concentration of BiTeI flakes (values greater than 82% were recorded for some devices). On the other hand, *J*_sc_ appears to be less sensitive to the BiTeI concentration ranging between 0.008 and 0.05 mg mL^−1^. In such a concentration range, the slight *J*_sc_ enhancement with the BiTeI concentration can be associated with the small bandgap of BiTeI (0.4 eV) that may allow light absorption at high wavelengths. By further increasing the concentration of the BiTeI flakes beyond 0.05 mg mL^−1^, the average *J*_sc_ drops from 20.56 to 20.15 mA cm^−2^, as shown in Fig. 2c.

**Fig. 3:**
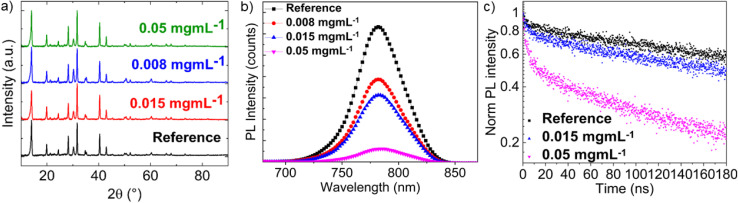
(a) XRD patterns, (b) PL spectra, and (c) Tr-PL *vs.* time plot of the pristine perovskite (reference) and BiTeI-incorporating perovskite layers, produced with a BiTeI concentration in the perovskite precursor solution of 0.008, 0.015 and 0.05 mg mL^−1^. The Tr-PL plot of the 0.008 mg mL^−1^ BiTeI-incorporating perovskite overlaps the data obtained for 0.015 mg mL^−1^ BiTeI concentration and was omitted for clarity.

In order to elucidate the mechanism behind the performance improvement of the cells upon incorporation of BiTeI flakes into the perovskite with the BiTeI flakes, additional device characterization was coupled with device simulations. Fig. 3a shows the XRD patterns of the pristine (reference sample) and BiTeI-incorporating perovskite layers, indicating that the addition of the BiTeI flakes into the perovskite absorber does not introduce any relevant structural/phase change of the perovskite. PbI_2_ was not observed in both reference and target samples. However, the full width at half maximum (FWHM) of the reflection (100) peak at 14° increases from 0.21 for the reference sample to about 0.25 for the samples produced with a BiTeI flake concentration in the perovskite precursor solution of 0.008 and 0.015 mg mL^−1^, and to 0.23 for the samples produced with a 0.05 mg mL^−1^ concentration of BiTeI additives. The trend of the FWHM of this peak can be associated with the perovskite strain or grain size change induced by the incorporation of the BiTeI flakes in the perovskite film. Fig. S1† shows the SEM images of the BiTeI-incorporated perovskite layers, compared to the pristine perovskite layer (reference sample). Clearly, the perovskite grains remain unchanged after the incorporation of BiTeI flakes into the perovskite layer, but as shown in Fig. S1b,† we observed bright areas scattered over the surface of the perovskite, likely indicating the presence of the BiTeI flakes. The increase in brightness is due to the higher conductivity of the BiTeI flakes compared to that of the perovskite. The large lateral size of BiTeI flakes excludes the possibility that they are incorporated into the perovskite crystal as a form of dopants. Based on the SEM image of Fig. S1,† the large BiTeI flakes are scattered over the perovskite surface, while the smaller ones could be located at the grain boundaries.

The charge carrier dynamics in the investigated perovskite layers was probed through PL and Tr-PL measurements. Fig. 3b shows the steady-state PL spectra of the pristine and BiTeI-incorporating perovskite layers. The PL peak is located at about 782 nm, as expected for our quadrupole-cation perovskite with a band gap of 1.58 eV. The perovskite bandgap was also confirmed by calculating the first derivative of the EQE spectrum, as shown in Fig. S2.† For the BiTeI-incorporating perovskites, the PL peak does not shift, but a PL quenching is observed as the BiTeI concentration increases. The Tr-PL measurements (Fig. 3c) reveal a drop of PL intensity during the first 20 ns, and this drop becomes more pronounced with increasing the concentration of the BiTeI flakes. Thus, the PL intensity has a dependence on the BiTeI concentration similar to *V*_oc_ (see Fig. 2d), that is, both the PL intensity and *V*_oc_ decrease with increasing the BiTeI concentration. Such a trend can be ascribed to a modification of the energy level alignment at the interface between the charge-extraction layers and the perovskite. To prove this mechanism, APS measurements were performed to evaluate the VB energy level and the *W*_F_ of the investigated perovskite layers (Fig. S3 and S4†). For all the perovskites, the *W*_F_ is *ca.* 5 eV, unaffected by the presence of the BiTeI flakes in the perovskite layer. In contrast to *W*_F_, the VB edge energy changes significantly by varying the concentration of BiTeI, as shown in Table 1. In particular, the energy level of the VB maximum of the perovskite shifts to deeper (more negative) levels upon the incorporation of the BiTeI flakes. The pristine perovskite has a VB maximum located at −5.43 eV, progressively shifting to lower values as the number of the BiTeI flakes increases (down to −5.66 eV). Table 1 also reports the energy level of the CB minimum of the perovskites, as estimated by the sum of the measured bandgap (1.58 eV) and the VB maximum energy. Overall, these results indicate a downward shift of the CB minimum and VB maximum energy levels of the perovskite absorbers with the incorporation of the BiTeI flakes.

**Table 1:** *W*_F_ and VB maximum energy level of the pristine perovskite and BiTeI-incorporating perovskites, estimated from APS measurements. The CB minimum energy level is also reported, as calculated from the sum of the bandgap (1.58 eV) and the VB maximum energy level of the perovskite. The CBO and VBO express the offset between the energy levels of the perovskite and the charge transporting layers and are calculated by using eqn (1) and (2). The table rows highlighted in green correspond to the BiTeI additive concentration that result in the optimal range of CB minimum energy level, CBO and VBO values. The rows highlighted in red correspond to the BiTeI additive concentrations that result in CB minimum energy level, CBO and VBO values worse than the optimal ones

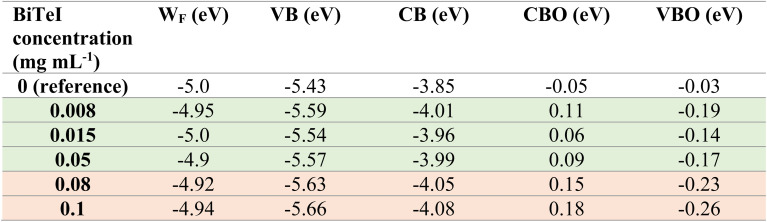

The response of the investigated PSCs was simulated using the SCAPS software.^77^ The input parameters for the simulations are listed in Tables S1–S3.† In particular, the CB minimum energy level of the perovskite was varied between −3.85 and −4.10 eV with a 0.05 eV step, to be consistent with the experimental values listed in Table 1. The simulation results are shown in Fig. 2, along with the experimental values (red curves and symbols). The simulations can reproduce the experimental trend observed in the *V*_oc_ and FF parameters. Specifically, the simulations indicate that the decrease of the CB minimum energy level: (1) considerably increases the device FF, (2) has a marginal influence on the device *J*_sc_, and (3) decreases the cell *V*_oc_ (for CB minimum energy level ≤ −4.0 eV). The combination of these effects leads to similar trends for the simulated and experimental PCEs, and a positive correlation is revealed between the CB minimum energy level and the device PCE until a certain threshold of the concentration of the BiTeI flakes in the perovskite precursor solution. In particular, upon the incorporation of the BiTeI flakes into the perovskite precursor solution with concentrations from 0.008 to 0.05 mg mL^−1^, the CB minimum energy level progressively decreases from −3.85 to around −4.00 eV, and the PCE of the corresponding devices becomes higher than that of the reference cell. However, by reaching a BiTeI concentration in the perovskite precursor solution equal to/greater than 0.08 mg mL^−1^, the CB minimum energy level is further lowered to −4.05 eV, while the PCE of the device starts to progressively degrade. The experiments and the simulations concur that the CB minimum energy values between −3.9 and −4.0 eV are beneficial for the device, while values lower than −4.05 eV cause a PCE drop.

**Fig. 4:**
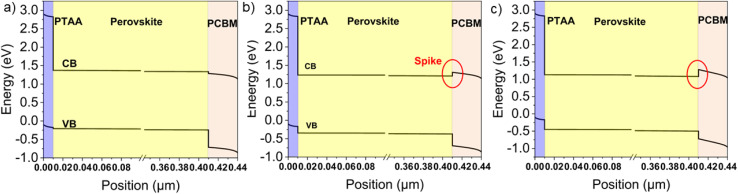
Simulated energy level diagrams of PSCs based on (a) a reference perovskite with a CB minimum energy level of −3.85 eV, and (b) a BiTeI-incorporating perovskite with a CB minimum energy level of −4.00 eV and (c) a BiTeI-incorporating perovskite with a CB minimum energy level of −4.10 eV. The energy levels are referenced to the equilibrium Fermi level in the device stack.

Fig. 4 shows the material energy level alignment across the device stack. The energy level alignment at the interfaces can be quantitively described by the conduction band offset (CBO) and valence band offset (VBO), *i.e.*, the energy difference between the CB minimum and VB maximum energy levels of the charge transporting layers and the perovskite, respectively. Thus, the CBO and VBO are obtained from the equations:

1 CBO = *χ*_perovskite_ − *χ*_PCBM_

2 VBO = *χ*_PTAA_ + *E*_g,PTAA_ − (*χ*_perovskite_ + *E*_g,perovskite_)

where *χ* stands for electron affinity and *E*_g,*x*_ for the bandgap of a material (denoted with *x*).

According to the above equations, for the reference device, the CBO is negative (−0.05 eV), meaning that the CB minimum energy level of the PCBM is more negative than that of the perovskite. Thus, at the perovskite/PCBM interface, the energy level diagram shows a “cliff” feature, and there is no energy barrier for the electron injection from the perovskite to the PCBM. Similarly, for the reference device, the VBO is −0.03 eV. This means that the VB maximum energy level of the PTAA is higher than that of the perovskite, and no energy barrier exists for the hole injection from the perovskite to the PTAA. When BiTeI flakes are added into the perovskite, the downward shift of the perovskite energy levels leads to a CBO reduction and eventually reverts its sign, while the VBO becomes even more negative. When the CBO is zero (that is, the CB minimum energy level of the perovskite is at −3.9 eV) the PCE increases, as shown by the experimental results and the simulation in Fig. 2a. A further downward shift in the energy levels of the perovskite leads to a positive CBO, *i.e.*, the CB minimum energy level of the PCBM being higher than that of the perovskite, leading to an electron injection barrier at the perovskite/PCBM interface. Consequently, the energy level diagram shows a “spike” feature. As shown in Fig. 4b, the “spike” feature at the perovskite/PCBM interface is formed when the CB minimum energy level of the perovskite drops to −4.00 eV, leading to a CBO of 0.1 eV and a VBO of −0.18 eV. In a first approximation, the alignment of the PTAA and PCBM energy levels with those of the perovskite seems to get worse upon any BiTeI doping concentration. However, the opposite trend was observed from both experimental results and simulations. Previous experimental work^85–89^ and simulations^90–94^ indicated that the PCE is maximized when there is a small positive CBO of 0.1 eV (“spike” configuration) and the VBO is found between −0.1 and −0.2 eV. Here, as shown in Table 1, after the addition of the BiTeI flakes into the perovskite solution with a concentration up to 0.05 mg mL^−1^, the CBO and VBO are within their optimal range (*i.e.*, 0 < CBO < 0.1 and −0.2 < VBO < −0.1). Consequently, the PCE of the corresponding devices is higher than that of the reference cells, in agreement with expectations. However, increasing further the BiTeI concentration, the CB minimum energy level becomes more negative than −4.05 eV, leading to CBO and VBO values higher than 0.1 eV and lower than −0.2 eV, respectively, as shown in Table 1 and Fig. 4c, thus causing PCE suppression.

**Fig. 5:**
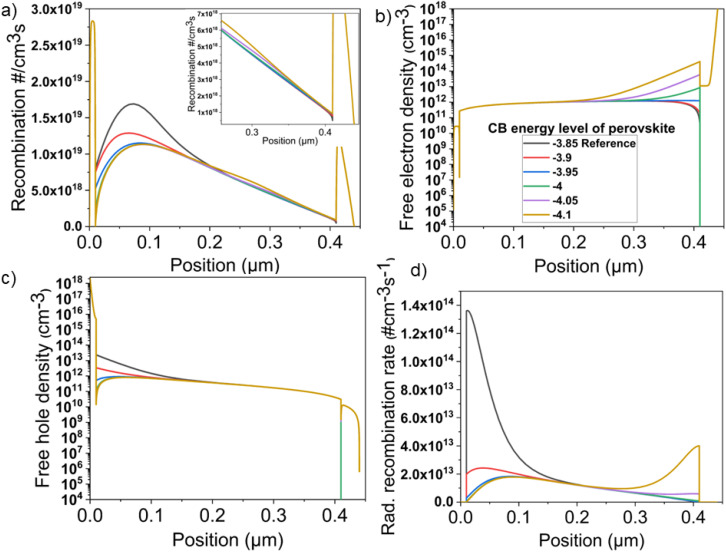
Device parameters extracted from the device simulation for PSCs based on pristine perovskite and BiTeI-incorporating perovskite, with CB minimum energy levels varying from −3.85 to −4.10 eV: (a) the recombination rate (the inset depicts a zoomed-in view near the PCBM region), (b) free electron density, (c) free hole density, and (d) radiative recombination rate across the device structure. The ITO contact is at *x* = 0, and *x* values increase as the rear metal contact approaches.

The above results evidence that the CBO and VBO values can affect the recombination rate at the interfaces of the perovskite with the charge transporting layers. Importantly, the rate of charge carrier generation is higher at the front of the device, and thus near the PTAA, leading, in turn, to a high recombination rate. This is well depicted in Fig. S4† and 5a, showing the charge carrier generation and recombination rate, respectively, across the device structure. The highest recombination rate for the reference device occurs near the first 100 nm of the perovskite layer, showing a maximum value of 1.69 × 10^19^ cm^−3^ s^−1^. The recombination rate drops for the CB minimum energy level of −3.9 eV, until reaching the lowest value of 1.14 × 10^19^ cm^−3^ s^−1^ when the VBO becomes close to −0.2 eV. The recombination rate near the perovskite/PTAA interface remains almost unchanged with lowering the CB minimum energy levels to less than −3.95 eV, even though a slightly higher recombination rate is observed in the middle region of the perovskite layer and close to the PCBM for a CB minimum energy level lower than −4.05 eV (inset of Fig. 5a), leading to lower device performance. Thus, an excessively negative CB minimum energy level concomitantly implies that the VB maximum energy level of the perovskite moves toward the highest occupied molecular orbital (HOMO) energy level of the PCBM, decreasing the carrier selectivity of the latter, *i.e.*, increasing the charge recombination rate (see Fig. 4c).

Fig. 5b and c show the free electron and hole densities across the device structure, respectively. The reference device exhibits a high density of free holes near the PTAA surface (distance < 0.1 μm), causing a high charge carrier recombination rate, as shown in Fig. 5a. The free hole density near the PTAA interface progressively decreases with decreasing the CB minimum energy level of the perovskite (Fig. 5c). In contrast, near the PCBM surface, the free electron density progressively increases with decreasing the CB minimum energy level of the perovskite (Fig. 5b). The high concentration of free carriers increases the rate of radiative recombination, as shown in Fig. 5d. As expected, the reference device exhibits the highest radiative recombination rate, located mostly near the PTAA surface, due to the high charge carrier generation rate and the free hole density. Radiative recombination is readily suppressed by lowering the CB minimum energies down to −4.05 eV. By further decreasing the CB minimum energy level, the increase of the free-electron concentration near the PCBM surface causes the enhancement of the radiative recombination near the PCBM surface. These results agree with our PL analysis (Fig. 3b), showing that the BiTeI addition into the perovskite effectively quenches the PL signal, explaining the experimental PCE trend.

## Discussion

Overall, our experiments and simulations reveal that the incorporation of BiTeI flakes into perovskite causes a downward shift of the perovskite energy levels. This, in turn, modifies the material energy level alignment at the perovskite/charge transporting layer interfaces. In particular, the positive and negative charges are optimally balanced across the device when the perovskite CB minimum energy level is between −3.90 and −4.00 eV. These values correspond to a VBO close to −0.2 eV and a CBO of about 0.1 eV, which result in optimal device PCE, as probed by *J*–*V* curve analysis (Fig. 2a). Moreover, the VBO value directly affects the charge carrier transporting properties across the perovskite/PTAA interface. In the reference device, the material energy levels at the PTAA/perovskite interface are not well aligned, while the presence of BiTeI in the perovskite leads to a better energy level alignment represented by optimal VBO values. The BiTeI-induced modification of the perovskite energy levels reduces the Shockley–Read–Hall (SHR) recombination and the radiative recombination near the perovskite/PTAA interface (Fig. 5a and d), improving the charge extraction, *i.e.*, enhancing device FF and PCE. In addition, the lowering of CB minimum energy level values of the perovskite upon BiTeI additive incorporation also improves the material energy level alignment at the perovskite/PCBM interface. When the CB minimum energy level of the perovskite is downshifted to the optimal range, namely between −3.90 and −4.00 eV, the CBO becomes positive and close to 0.1 eV. Under these CB minimum energy level conditions, the energy level diagram reveals a “spike” feature at the perovskite/PCBM interface that has a beneficial impact on carriers' transporting properties. It was shown that the “cliff” configuration blocks the flow of injected electrons from the buffer layer to the absorber under forward bias and thus, the accumulation of injected electrons can increase the interface recombination current at the heterojunction interface.^95^ This was quantitatively shown in ref. ^96^, in which the “spike” band alignment results in lower interface recombination. Therefore, the “spike” feature reduces the surface recombination of electrons with free holes from the perovskite layer at the perovskite/PCBM interface (Fig. 5b).^85,89,97^ However, an excess of BiTeI flakes in the perovskite lowers too much the CB minimum energy level, causing a progressive suppression of the device performance. In particular, a CB minimum energy level lower than −4.05 eV results in an unfavorable energy level alignment at the interfaces, as described by the non-optimal VBO and CBO values (*i.e.*, CBO > 0.1 and VBO < −0.2). Briefly, CBO values higher than 0.1 eV translate into a higher injection barrier for the electrons, decreasing the electron extraction rate. Optimal CB minimum energy level values range from −3.90 eV to −4.0 eV, as those achieved in the BiTeI-incorporating perovskite produced with BiTeI concentrations in the perovskite precursor solution of 0.008, 0.015 and 0.05 mg mL^−1^. It is noted that the simulations cannot account for the full physical mechanism behind the insertion of the BiTeI flakes into the perovskite. For example, the BiTeI flakes have a small direct bandgap of about 0.4 eV. This means that they can absorb light in the infrared (IR) spectral region, possibly increasing the device *J*_sc_, as shown in Fig. 2c. Nevertheless, our simplified approach, simulating the effect of CB minimum energy level variance, avoids overparameterization of the system, while displaying good matching between the experimental and simulated performance trends. Besides, this is justified by our data, which support that the CB minimum energy level is a crucial factor, strongly affecting the device performance.

## Conclusions

This study has identified that material energy level misalignment at PTAA/perovskite and perovskite/PCBM interfaces can limit the performance of inverted PSCs. Optimal energy level alignment has been achieved by incorporating LPE-produced 2D BiTeI flakes into the perovskite layer, at optimal BiTeI concentration in the perovskite precursor solution between 0.008 and 0.05 mg mL^−1^. The presence of BiTeI flakes in the perovskite confers a n-type behavior to the perovskite, inducing a downshift of the CB minimum and VB maximum energy levels of about 0.10–0.25 eV. By combining simulations with our experimental data, we demonstrate that when the VBO and CBO are close to −0.2 and 0.1 eV, respectively, the device FF is greatly improved, while the *V*_oc_ remains unchanged, leading to an overall PCE improvement. The analysis of the results indicates that the “spike” feature of 0.1 eV located at the perovskite/PCBM interface is beneficial for the device performance. However, when the CB minimum energy level of the perovskite is lower than −4.05 eV, the VB maximum energy level of the perovskite shifts far away from the HOMO energy level of the PTAA, while approaching that of PCBM's HOMO, causing a drop of the device *V*_oc_. Moreover, the injection barrier for electrons increases at the perovskite/PCBM interface, reducing the electron extraction rate. The optimization of the energy level alignment at the interfaces between the perovskite and the charge transporting layers improves the distribution of the charge carriers across the device, reducing the overall charge carrier recombination rate and improving the charge carrier extraction from the perovskite towards the charge transporting layers. By optimizing the BiTeI additive concentration, the PSC based on a BiTeI-incorporating perovskite exhibits a significant improvement of its FF compared to control devices, reaching values higher than 82%. Consequently, our best BiTeI-based PSCs delivered a PCE of 18.3%, with an average PCE showing a +8% increase compared to the control devices. Overall, these findings demonstrate that 2D BiTeI flakes constitute an effective additive for the perovskite, aimed at a simple and effective way to increase the performance of inverted PSCs.

## Conflicts of interest

There are no conflicts of interest to declare.

## Supplementary Material

SE-006-D2SE01109C-s001
