# Supplementary material for: Two-dimensional BiTeI as a novel perovskite additive for printable perovskite solar cells
Source: Sustain Energy Fuels. 2022 Oct 24;6(23):5345–59. doi: 10.1039/d2se01109c (PMC9907396; doi:10.1039/d2se01109c)
Supplement: SE-006-D2SE01109C-s001 [file SE-006-D2SE01109C-s001.pdf]

Electronic Supplementary Material (ESI) for Sustainable Energy & Fuels.
This journal is © The Royal Society of Chemistry 2022

Supplementary Information

Two-Dimensional BiTeI as a Novel Perovskite Additive for Printable Perovskite Solar Cells

Dimitris Tsikritzis[1,2], Konstantinos Chatzimanolis[1], Nikolaos Tzoganakis[1], Sebastiano Bellani[3], Marilena Isabella Zappia[3], Gabriele Bianca[4], Nicola Curreli[5], Joka Buha[3,6], Ilka Kriegel[5], Nikolas Antonatos[7], Zdeněk Sofer[7], Miron Krassas[1], Konstantinos Rogdakis,[1,2] Francesco Bonaccorso[3,4] and Emmanuel Kymakis[1,2]

[1]Department of Electrical & Computer Engineering, Hellenic Mediterranean University (HMU), Heraklion 71410, Crete, Greece.

[2] Institute of Emerging Technologies (i-EMERGE) of HMU Research Center, Heraklion 71410, Crete, Greece

[3] BeDimensional S.p.A., Via Lungotorrente secca 30R, 16163 Genova, Italy

[4] Graphene Labs, Istituto Italiano di Tecnologia, via Morego, 30, 16163, Genova, Italy

[5] Functional Nanosystems, Istituto Italiano di Tecnologia, via Morego, 30, 16163, Genova, Italy

[6] Department of Nanochemistry, Istituto Italiano di Tecnologia, via Morego, 30, 16163, Genova, Italy

[7] Department of Inorganic Chemistry, University of Chemistry and Technology Prague, Technická 5, Prague 6, 16628 Czech Republic

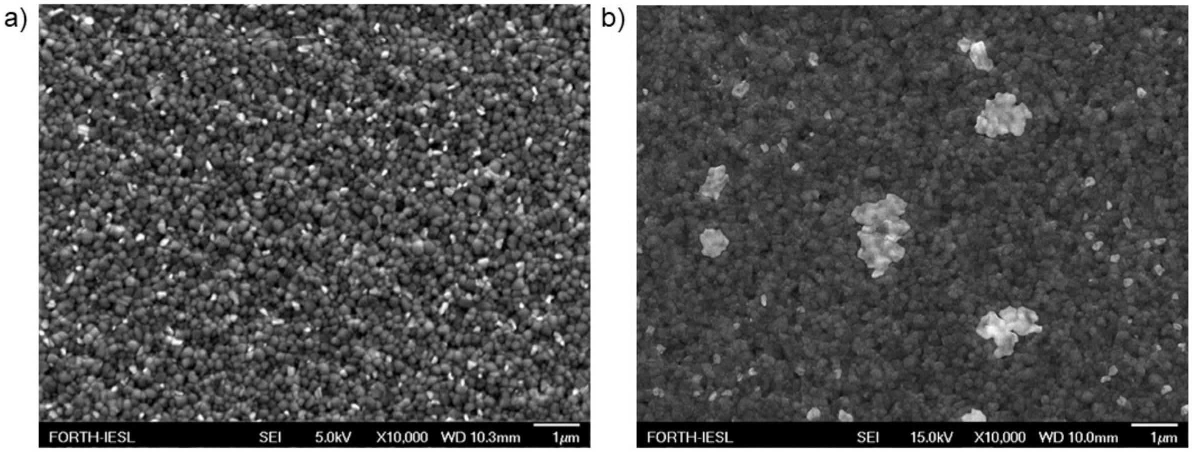

Figure S1. Top-view SEM images of the a) pristine perovskite and b) BiTeI-incorporating perovskite produced with a BiTeI flakes concentration in the perovskite precursor solution of 0.05 mg mL^{-1}.

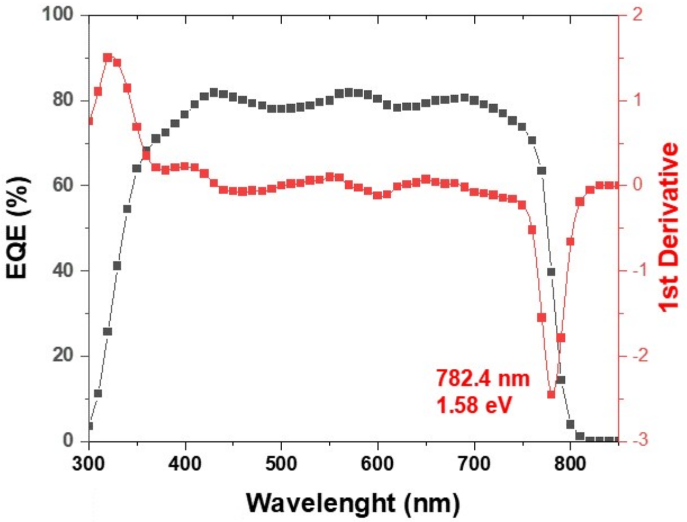

Figure S2. EQE spectrum and its first derivative measured for the PSC based on the BiTeI-incorporating perovskite produced with a BiTeI flakes concentration in the perovskite precursor solution of the 0.05 mg mL^{-1}. The minimum of the first derivative corresponds to the photovoltaic device bandgap of the perovskite.

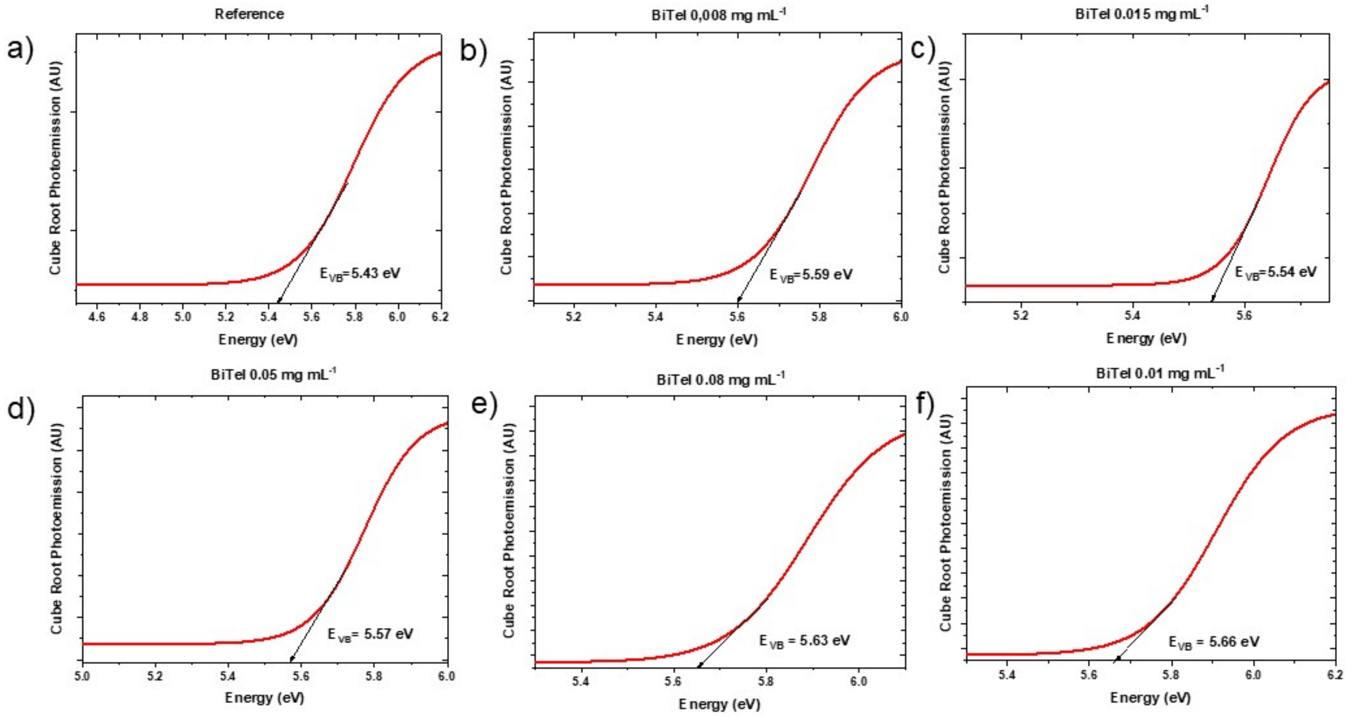

Figure S3. Ambient photoemission spectroscopy spectra measured for a) the pristine perovskite, and the BiTeI-incorporating perovskite produced with a BiTeI flakes concentration in the perovskite precursor solution of b) 0.008 mg mL^{-1}, c) 0.015 mg mL^{-1}, d) 0.05 mg mL^{-1}, e) 0.08 mg mL^{-1}, and f) 0.1 mg mL^{-1}. The spectra have been used to estimate the VB maximum energy levels of the investigated perovskites.

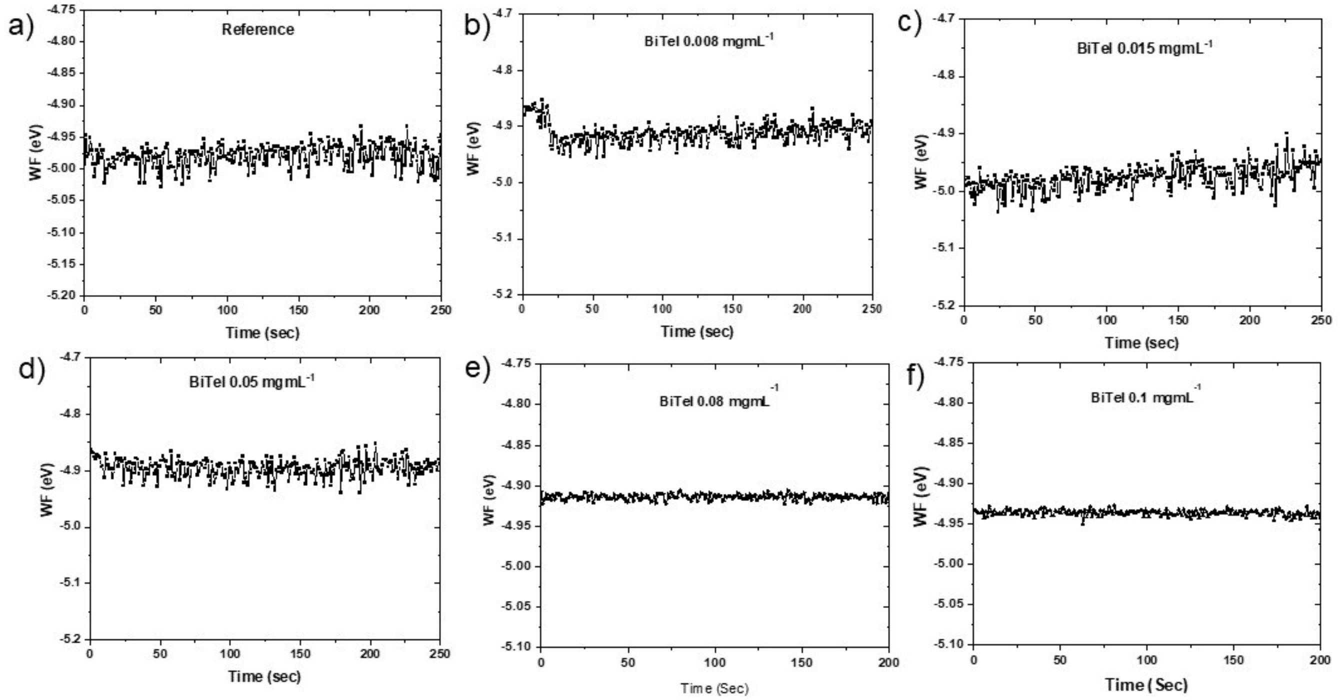

Figure S4. WF measurements for a) measured for a) the pristine perovskite, and the BiTeI-incorporating perovskites produced with a BiTeI flakes concentration in the perovskite precursor solution of b) 0.008 mg mL^{-1}, c) 0.015 mg ml^{-1}, d) 0.05 mg mL^{-1}, e) 0.08 mg mL^{-1}, and f) 0.1 mg mL^{-1}.

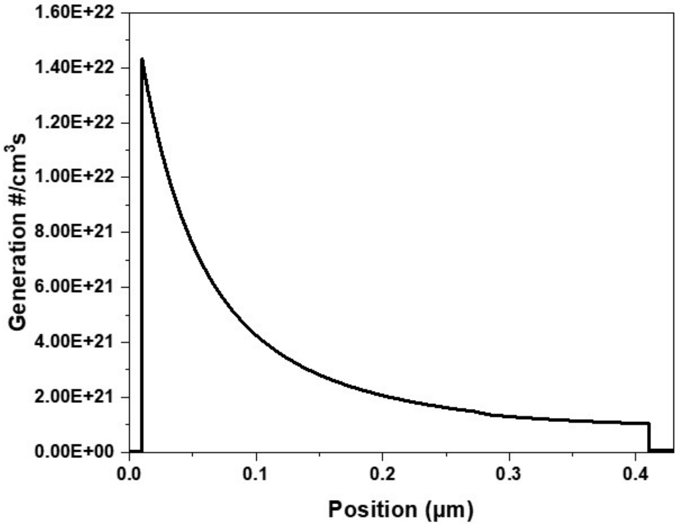

Figure S5. Simulation of the charge carrier generation rate across the device. The charge carrier generation rate in the perovskite increases near the perovskite/PTAA interface, while it is suppressed near the perovskite/PCBM interface. The generation rate is the same for the pristine and BiTeI incorporating devices.

Table S1. Materials properties of PTAA, perovskite, and PCBM used in the device simulations.

Parameter	**PTAA**	**Perovskite**	**PCBM**
Bandgap (eV)	3.0	1.58	2
Electron Affinity	2.4	3.85-4.1	3.9
Dielectric permittivity	3.5	70	3
Thickness (nm)	10	400	30
CB/VB effective density of states (cm^{-3})	$1x10^{20}$	$1.5x10^{18}$	$1x10^{20}$
Thermal velocity e^-/h^+	$1x10^7$	$1x10^7$	$1x10^7$
Electron mobility ($cm^2V^{-1}s^{-1}$)	$1.5x10^{-4}$	10	$5x10^{-2}$
Hole mobility ($cm^2V^{-1}s^{-1}$)	$1.5x10^{-4}$	10	$5x10^{-2}$
Shallow donor density (cm^{-3})	0	$1x10^{13}$	$1x10^{15}$
Shallow acceptor density (cm^{-3})	$1x10^{15}$	$1x10^{13}$	0
Radiative recombination coefficient	0	$3x10^{-11}$	0
Absorption	sqrt(hv-E_g) law, a_0=$1x10^4$, b^0=$1x10^{-12}$	From [1]	sqrt(hv-E_g) law, a_0=$1x10^5$, b^0=$1x10^{-12}$

Table S2. Properties related to defects in PTAA, perovskite, PCBM, and interfaces used in device simulations.

	PTAA	Perovskite	PCBM	Perovskite/PTAA	Perovskite/PCBM
Defect type	Neutral	Neutral	Neutral	Neutral	Neutral
capture cross section electrons and holes (cm^{-2})	$5x10^{-14}$	$1x10^{-14}$	$5x10^{-14}$	$1x10^{-19}$	$1x10^{-19}$
Energetic Distribution	Gauss	Gauss	Gauss	Gauss	Gauss
characteristic	0.1	0.1	0.1	0.1	0.1

energy (eV)					
Defect Energy Position (eV)	0 above Ei	0 above Ei	0 above Ei	0 above Ei	0 above Ei
Defect density (cm^{-3})	$2x10^{15}$	$3x10^{14}$	$2x10^{15}$	$2x10^{14}$	$1x10^{15}$
Lifetime (ns)	1	33	1		
Diffusion Length (μm)	$6.2x10^{-4}$	0.93	$1.1x10^{-2}$		
Surface recombination velocity (cms^{-1})	0	0	0	200	1000

Table S3. Properties of contacts used in device simulation.

Parameter	ITO	Ag	Device
Thermionic emission e^-/h^+ (cm s^{-1})	$1x10^7$	$1x10^7$	
Majority carrier barrier height relative to the E_F (eV)	0.1	0.1	
Light Transmission	0.85		
Light Reflection		0.50	
Series Resistance (Ωcm^2)			1*

* The simulation of devices based on BiTeI-incorporating perovskite resulted in FF values over 84%. To suppress FF close to the experimental values, we considered the addition of a series resistance of 1 Ω cm^2 for simulating the d devices based on the BiTeI-incorporating perovskites.

The input parameters for the simulations were acquired from experimental results and the literature [2]–[7].

References

[1] A. Tejada *et al.*, “Hybrid Perovskite Degradation from an Optical Perspective: A Spectroscopic Ellipsometry Study from the Deep Ultraviolet to the Middle Infrared,” *Adv. Opt. Mater.*, vol. 10, no. 3, p. 2101553, Feb. 2022, doi: 10.1002/adom.202101553.

[2] M. Stolterfoht, V. M. Le Corre, M. Feuerstein, P. Caprioglio, L. J. A. Koster, and D. Neher, “Voltage-Dependent Photoluminescence and How It Correlates with the Fill Factor and Open-Circuit Voltage in Perovskite Solar Cells,” *ACS Energy Lett.*, vol. 4, no. 12, pp. 2887–2892, Dec. 2019, doi: 10.1021/acsenergylett.9b02262.

[3] J. Diekmann *et al.*, “Pathways toward 30% Efficient Single-Junction Perovskite Solar Cells and the Role of Mobile Ions,” *Sol. RRL*, vol. 5, no. 8, p. 2100219, Aug. 2021, doi: 10.1002/solr.202100219.

[4] T. Minemoto, Y. Kawano, T. Nishimura, and J. Chantana, “Numerical reproduction of a perovskite solar cell by device simulation considering band gap grading,” *Opt. Mater. (Amst).*, vol. 92, no. December 2018, pp. 60–66, Jun. 2019, doi: 10.1016/j.optmat.2019.03.048.

[5] T. Minemoto and M. Murata, “Theoretical analysis on effect of band offsets in perovskite solar cells,” *Sol. Energy Mater. Sol. Cells*, vol. 133, pp. 8–14, Feb. 2015, doi: 10.1016/j.solmat.2014.10.036.

[6] R. Brenes *et al.*, “Metal Halide Perovskite Polycrystalline Films Exhibiting Properties of Single Crystals,” *Joule*, vol. 1, no. 1, pp. 155–167, 2017, doi: 10.1016/j.joule.2017.08.006.

[7] P. Zhao *et al.*, “Device simulation of inverted CH3NH3PbI3−xClx perovskite solar cells based on PCBM electron transport layer and NiO hole transport layer,” *Sol. Energy*, vol. 169, no. April, pp. 11–18, Jul. 2018, doi: 10.1016/j.solener.2018.04.027.
